# Supplementary material for: Molecular structure characterization analysis and molecular model construction of anthracite
Source: PLoS One. 2022 Sep 28;17(9):e0275108. doi: 10.1371/journal.pone.0275108 (PMC9703969; doi:10.1371/journal.pone.0275108)
Supplement: Attachment — Submitted filename: Response to Reviewers.docx [file pone.0275108.s001.docx]

Dear editor:

Thank you very much for giving us an opportunity to revise our manuscript. We appreciate the editor and reviewers very much for their constructive comments and suggestions on our manuscript entitled“**Molecular structure characterization analysis and molecular model construction of anthracite**”(ID: PONE-D-22-16377).

We have studied reviewers' comments carefully. According to the reviewers' detailed suggestions. we have made a careful revision on the original manuscript. I modified the copy of the markup to form a separate file, named 'Revised Manuscript with Track Changes'.

Kind regards.

Corresponding author: WU Yumo

E-mail address: 13614067811@163.com

Replies to the reviewers’ comments:

Reviewer #1:

1. (i) x-axis of IR spectra is wrong

(ii) y-axis of XRD pattern is wrong

(iii) improve the quality of figures

Response: We feel sorry for our carelessness. In our resubmitted manuscript, the typo is revised, and upload the modified image as a separate file. Thanks for your correction.

Reviewer #2:

1. The authors have used elemental analysis to understand the CHNO content C208 H162 O12 N4 is matching with the proposed structure (I took the pain of drawing the whole structure to compare the molecular structure). however, the ultimate analysis shows very less H content (85.43:4.52:6.53:1.98). why these discrepancies? and also please shine a light on proximate and ultimate analysis.

Response: We sincerely thank the reviewer for careful reading. In the structural model, the red-labeled H is only shown in the hydroxyl group, while the remaining H is not shown in the benzene ring and other structures.

2. Kindly add the following reference “New advances in coal Structural model” Zhihong, Q., International journal of mining science and technology, 2018, 541-559.

Response: We sincerely appreciate the valuable comments. As suggested by the reviewer, we have added more references to support this idea (New advances in coal Structural model). According to literature 36, our model building has played a guiding role.
